# Exposure to recurrent hypoglycemia alters hippocampal metabolism in treated streptozotocin‐induced diabetic rats

**DOI:** 10.1111/cns.13186

**Published:** 2019-07-07

**Authors:** Neelesh Dewan, Vibha Shukla, Ashish K. Rehni, Kevin B. Koronowski, Kyle D. Klingbeil, Holly Stradecki‐Cohan, Timothy J. Garrett, Tatjana Rundek, Miguel A. Perez‐Pinzon, Kunjan R. Dave

**Affiliations:** ^1^ Peritz Scheinberg Cerebral Vascular Disease Research Laboratories University of Miami School of Medicine Miami Florida USA; ^2^ Department of Neurology University of Miami School of Medicine Miami Florida USA; ^3^ Neuroscience Program University of Miami School of Medicine Miami Florida USA; ^4^ Southeast Center for Integrated Metabolomics, Clinical and Translational Science Institute University of Florida Gainesville Florida USA; ^5^ Evelyn F. McKnight Brain Institute University of Miami School of Medicine Miami Florida USA

**Keywords:** diabetes, enzyme kinetics, glycolysis, hippocampus, metabolomics

## Abstract

**Aims:**

Exposure to recurrent hypoglycemia (RH) is common in diabetic patients receiving glucose‐lowering therapies and is implicated in causing cognitive impairments. Despite the significant effect of RH on hippocampal function, the underlying mechanisms are currently unknown. Our goal was to determine the effect of RH exposure on hippocampal metabolism in treated streptozotocin‐diabetic rats.

**Methods:**

Hyperglycemia was corrected by insulin pellet implantation. Insulin‐treated diabetic (ITD) rats were exposed to mild/moderate RH once a day for 5 consecutive days.

**Results:**

The effect of RH on hippocampal metabolism revealed 65 significantly altered metabolites in the RH group compared with controls. Several significant differences in metabolite levels belonging to major pathways (eg, Krebs cycle, gluconeogenesis, and amino acid metabolism) were discovered in RH‐exposed ITD rats when compared to a control group. Key glycolytic enzymes including hexokinase, phosphofructokinase, and pyruvate kinase were affected by RH exposure.

**Conclusion:**

Our results demonstrate that the exposure to RH leads to metabolomics alterations in the hippocampus of insulin‐treated streptozotocin‐diabetic rats. Understanding how RH affects hippocampal metabolism may help attenuate the adverse effects of RH on hippocampal functions.

## 1 INTRODUCTION

Hypoglycemia remains a significant barrier to achieving adequate glycemic control in patients with diabetes mellitus.^1^ Repeated exposure to hypoglycemia results in defective glucose counterregulation leading to impaired hypoglycemia awareness.^2^, ^3^ Incidences of both severe and asymptomatic hypoglycemia are reported frequently in type 1 and type 2 diabetes mellitus (T1DM and T2DM) patients.^3^, ^4^, ^5^, ^6^, ^7^, ^8^, ^9^, ^10^, ^11^ An earlier study from Boland et al, that monitored T1DM patients using a continuous glucose monitoring system, reported that T1DM patients frequently experience prolonged and asymptomatic hypoglycemia (glucose < 60 mg/dl).^12^ A similar study from Gehlaut et al in T2DM patients also reported exposure to frequent asymptomatic hypoglycemia (glucose < 70 mg/dl).^6^

Neuronal damage and death occurs in the hippocampus following exposure to severe hypoglycemia, and in the prefrontal, orbital, and piriform cortex following moderate hypoglycemia.^13^, ^14^ In rat models, exposure to RH increases oxidative damage to hippocampal neurons, as well as microglial activation leading to chronic cognitive impairment.^15^ Previous animal studies examining spatial working memory and hippocampal function reported that RH enhances spatial working memory in rats in euglycemia but impairs it during subsequent hypoglycemic episodes.^16^, ^17^ Reports also demonstrate increased anxiety in previously RH‐exposed rats during acute hypoglycemia and decreased mental flexibility when euglycemic.^18^ Furthermore, the hippocampal synaptic function was found to be markedly impaired in RH‐exposed rats during further hypoglycemic conditions.^17^ Human studies are controversial; difficulties in controlling for diabetic history and related disease processes have produced mixed results. The Diabetes Control and Complications Trial (DCCT), and later the Epidemiology of Diabetes Interventions and Complications (EDIC) follow‐up study, concluded that the frequency of severe hypoglycemia was not related to subsequent cognitive impairment.^19^ Another recent study however observed that exposure to hypoglycemia correlates with worsening cognitive deficits, including impairments of learning and memory.^20^ In addition, the level of cognitive impairment, as a result of acute hypoglycemia in potentially hippocampus‐mediated complex tasks (eg, driving), has been related to the severity of prior hypoglycemic episodes.^21^

During normal glycemic periods, the human brain mainly depends on the systemic supply of glucose as the principal metabolic substrate.^22^, ^23^ Exposure to hypoglycemia leads to several metabolic adaptations such as increased levels of glucose transporters, subsequently enhanced uptake of glucose, increase in brain glucose levels, increased levels of hexokinase I, increased glucose phosphorylation, and increased glycolytic flux in vitro*.* 24, 25, 26, 27, 28, 29, 30, 31, 32, 33 Besides, exposure to aglycemia and/or RH also affects the tricarboxylic acid (TCA) cycle in multiple ways.^32^, ^34^, ^35^ Therefore, the literature shows differential effects of hypoglycemia on various pathways of cellular metabolism. However, the global effect of RH exposure on the brain metabolome is not known. Understanding the effects of hypoglycemia on global brain metabolism may also help us understand the pathological consequences of hypoglycemia on important brain structures involved in cognitive functions such as the hippocampus. The primary objective of this study is to investigate the effects of mild‐to‐moderate hypoglycemia on hippocampal metabolism. We hypothesize that exposure to RH leads to metabolomic alterations in the hippocampus of insulin‐treated streptozotocin‐diabetic rats.

## 2 MATERIALS AND METHODS

### 2.1 Induction of diabetes, insulin treatment, and induction of recurrent hypoglycemia

**Figure 1:**
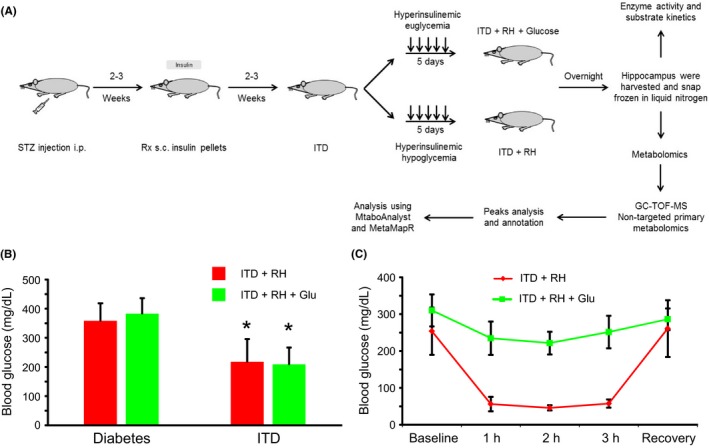
A, Schematic diagram of the experimental paradigm. B, Blood glucose levels after streptozotocin injection, before and after insulin pellet implantation. Blood glucose levels measured for ITD + RH + glucose (n = 16) and ITD + RH (n = 16) groups after streptozotocin injection (ie, before insulin pellet implantation) and following insulin pellet implantation (at the time of tissue harvest). C, Blood glucose levels during hypoglycemia for ITD + RH + glucose (n = 16) and ITD + RH (n = 16) groups. *P < 0.05 vs pre‐insulin pellet implantation (ie, untreated diabetes)

All experimental procedures were carried out as per the Guide for the Care and Use of Laboratory Animals published by the National Institutes of Health and in accordance with the protocols approved by the Animal Care and Use Committee of the University of Miami. Results are reported according to the ARRIVE guidelines to the best of our ability. Rats were made diabetic by injecting the pancreatic β‐cell toxin streptozotocin.^36^ The rats having blood glucose values ≥250 mg/dl were included in the diabetic group. Insulin pellets were implanted subcutaneously (sc) after 2‐3 weeks of diabetes induction.^36^ This group of animals was considered as insulin‐treated diabetic (ITD) rats. ITD group rats were randomly divided into ITD + RH (n = 10) and ITD + RH + glucose (n = 10) groups. Rats belonging to ITD + RH or ITD + RH + glucose groups were exposed to five episodes of RH (hyperinsulinemic hypoglycemia) or RH + glucose (hyperinsulinemic euglycemia) over five consecutive days (1 episode/day), respectively (see Appendix S1 for details). One day after the last hypoglycemic exposure, the rats were euthanized by decapitation under isoflurane anesthesia (30% O_2_/70% N_2_O); brains were quickly excised; hippocampi were harvested, snap‐frozen in liquid nitrogen, and stored at −80°C until use (Figure 1A).

### 2.2 Metabolomic and enzyme studies

Samples were analyzed in a blinded manner. The sequence of analysis was randomized using a random number generator to select the run order. Metabolomic data were analyzed using MetaboAnalyst 3.0 software.^37^ Connections between related metabolites were generated using MetaMapR in order to build a network that displays structural similarity based on a metabolite's PubChem substructure fingerprints.^38^ The hippocampus was thawed on ice and homogenized, and its supernatant was collected and enzymatic assays were performed following the manufacturer's instructions. Refer Appendix S1 for details.

### 2.3 Statistical analysis

Comparison of the two groups was performed using the *Student's t test* for all parameters. Significant outlier data points, if any, as identified by Grubbs’ test were excluded from further analysis. The results are presented as mean ± SD. A *P*‐value of <0.05 was considered statistically significant. Statistical methods used for analyzing metabolomics results are provided in the metabolomics methods section above.

## 3 RESULTS

### 3.1 Glucose levels following induction of diabetes, during insulin treatment, and hypoglycemia

The experimental procedure is presented in Figure 1A. The results show that the glucose levels prior to insulin treatment in RH‐exposed ITD animals and ITD + RH + glucose rats were not statistically different. Similarly, there was no significant difference between the blood glucose levels measured after insulin treatment in ITD + RH and ITD + RH + glucose groups (Figure 1B). To avoid unwanted hypoglycemia, blood glucose levels during insulin treatment were kept slightly above euglycemic levels. The mean blood glucose levels during induction of hypoglycemia (during hyperinsulinemic hypoglycemia) in the ITD + RH group were 53 ± 14 mg/dl. These values were significantly lower than the blood glucose levels observed in ITD + RH + glucose (during hyperinsulinemic euglycemia) (236 ± 42 mg/dl) (Figure 1C).

### 3.2 Exposure to recurrent hypoglycemia alters hippocampal metabolic pathways

**Figure 2:**
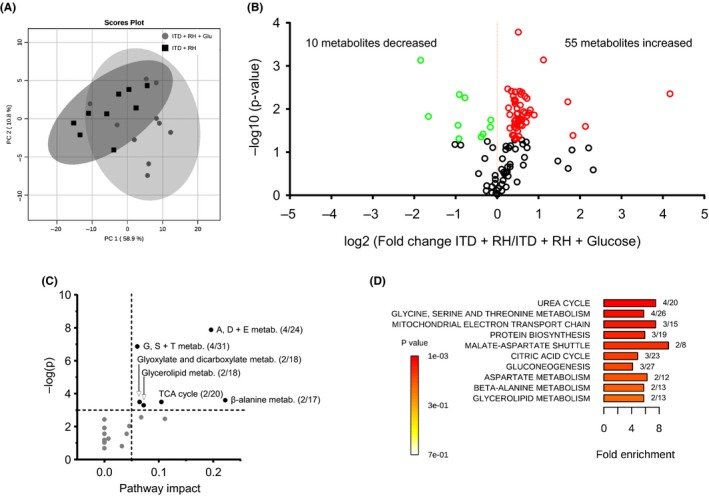
The effect of exposure to hypoglycemia to ITD rats on hippocampal metabolic pathways. A, Scores plot from principal component analysis (PCA) showing clustering of groups based on quantitative variances of annotated metabolites. 95% confidence intervals are displayed for each group. B, Volcano plot of –log(*P*‐value) and log2(fold‐change) values for all annotated metabolites. Metabolites that were significantly higher (red), lower (green), or not different (black open circles) in ITD + RH group as compared to ITD + RH + glucose group. C, Pathway and enrichment analysis for significantly altered metabolites in ITD + RH group as compared to ITD + RH + glucose group. Higher pathway impact value indicates greater centrality or importance in that particular pathway. Dashed lines display significance cutoffs (see text). G, S + T = glycine, serine and threonine; A, D + E = alanine, aspartate and glutamate. D, Enrichment analysis for metabolite sets based on normal metabolic function. Fold enrichment values indicate whether metabolites appear more than expected by random chance (see text for significance cutoffs). Ratios presented next to each bar denote the number of metabolites identified vs the total number of metabolites in that pathway. For example, 4/20 presented next to the urea cycle bar means that 4 metabolites were identified in that pathway of 20 total metabolites

Here, we evaluated the effect of RH exposure in ITD rats on hippocampal metabolism by nontargeted, global metabolite profiling. In total, 386 peaks were captured, 115 of which are structurally annotated metabolites. Basal metabolic differences and putative RH‐regulated pathways were identified by comparing ITD + RH (n = 9) and the control group (ITD + RH + glucose, n = 10). The principal component analysis revealed some separation between the groups along principal component 1 (PC1, 58.9% of total variance), indicating a small overall difference between metabolite profiles of ITD + RH and ITD + RH + glucose groups (Figure 2A). Comparison of individual metabolites found 65 significantly altered metabolites in the ITD + RH group (n = 9) compared with the ITD + RH + glucose group (n = 10) (Student's *t* test, *P* < 0.05, false discovery rate: FDR < 0.1, Figure 2B and Table S1). Of these 65 metabolites, the majority were increased (55 metabolites), while only a small fraction was decreased in abundance (10 metabolites).

**Figure 3:**
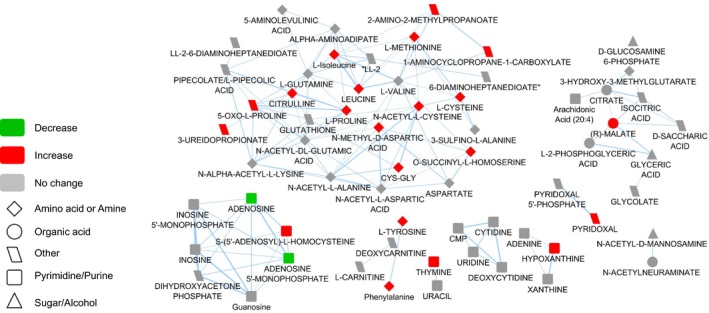
Structural similarity network displaying metabolic perturbations in hippocampus of RH‐exposed ITD rats. Metabolites are linked based on structural similarity of PubChem substructure fingerprints. Shape denotes chemical class from KEGG Compound. Relative abundance in ITD + RH is represented by color, with respect to ITD + RH + glucose. This graphic serves as a comprehensive visualization of the identified metabolic changes

**Figure 4:**
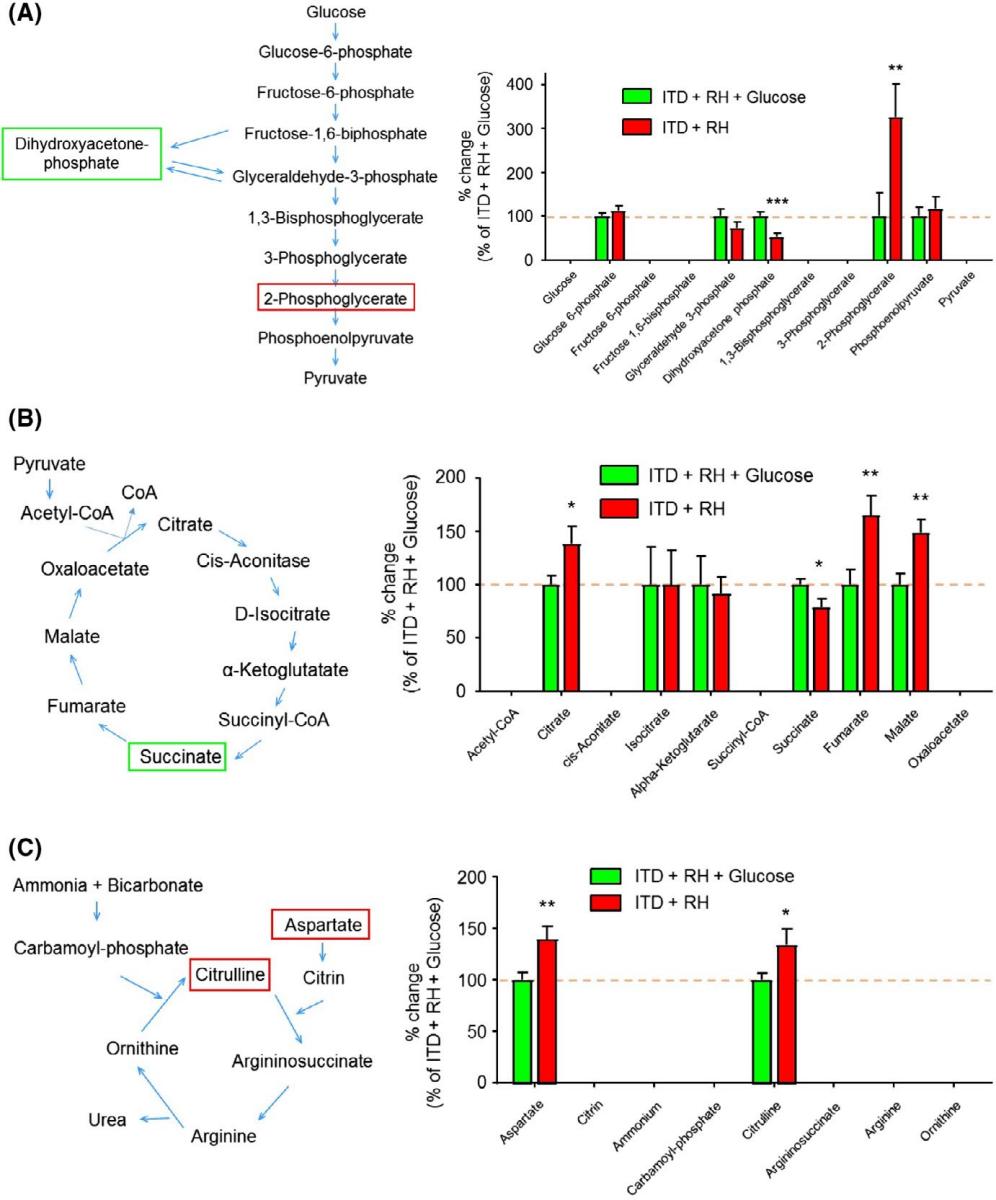
The hippocampal levels of (A) glycolysis, (B) citric acid, and (C) urea cycle intermediate in ITD + RH + glucose (n = 10) and ITD + RH (n = 9) groups. It should be noted that the values for some of the metabolites are not presented on bar graphs as either our method was not been able to detect levels of those missing metabolites or those metabolites are not yet annotated. *P < 0.05, **P < 0.01, and ***P < 0.005

Pathway analysis using the KEGG pathway database identified six significantly altered pathways in the hippocampus of RH‐exposed ITD rats, when compared to the ITD + RH + glucose group: (a) alanine, aspartate, and glutamate metabolism (aspartic acid, alanine, fumaric acid, glucosamine 6‐phosphate); (b) glycine, serine, and threonine metabolism (glyceric acid, betanin, sarcosine, threonine); (c) β‐Alanine metabolism (β‐alanine, aspartic acid, ureidopropionic acid); (d) glyoxylate and dicarboxylate metabolism (glyceric acid and malic acid); (e) glycerolipid metabolism (glyceric acid dihydroxyacetone phosphate); and (f) citrate cycle (malic acid, fumaric acid) (≥2 hits, impact > 0.05, *P* ≤ 0.01, Figure 2C). Enrichment analysis also identified several other significantly altered pathways (≥2 hits, fold enrichment ≥ 2.0, *P* ≤ 0.01, Figure 2D). Ratios presented next to each bar in Figure 2D denote the number of metabolites identified vs the total number of metabolites in that pathway. These include the urea cycle (fumaric acid, alanine, aspartic acid, ADP); the mitochondrial electron transport chain (fumaric acid, ADP, dihydroxyacetone phosphate); protein biosynthesis (alanine, threonine, aspartic acid); malate‐aspartate shuttle (aspartic acid, malic acid); and gluconeogenesis (malic acid, ADP, dihydroxyacetone phosphate). Structural similarity networks analysis indicated that there are several chemical classes of metabolites more abundant in the hippocampus of RH‐exposed ITD rats (Figure 3, red‐colored shapes). These chemical classes include amino acid/amine, organic acid, and purine/pyrimidine. Altered metabolites belonging to glycolysis, the citric acid cycle, and the urea cycle are presented in Figure 4A‐C, respectively. Also, decreases in several metabolites belonging to the pyrimidine/purine chemical class were observed. Overall, our results indicate that exposure to RH affects several metabolic pathways in the hippocampus of ITD rats.

### 3.3 Substrate kinetics of key glycolytic enzymes

**Figure 5:**
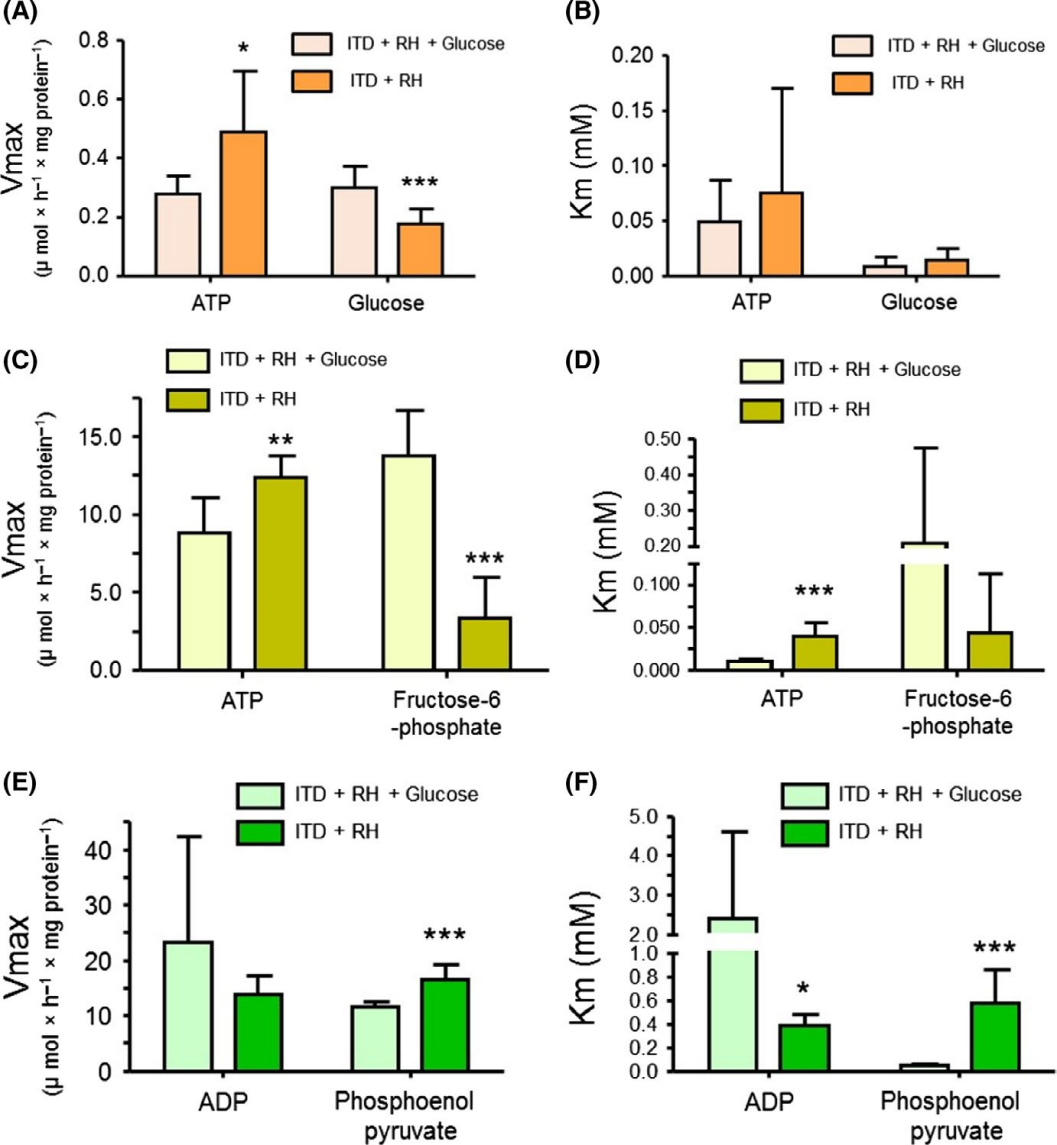
A, B, The substrate kinetic properties (*V*_max_ and *K*_m_) of hippocampal hexokinase in ITD + RH + glucose (n = 6) and ITD + RH (n = 7) groups. C, D, The substrate kinetic properties (*V*_max_ and *K*_m_) of hippocampal phosphofructokinase in ITD + RH + glucose (n = 6) and ITD + RH (n = 5–6) groups. E, F, The substrate kinetic properties (Vmax and Km) of hippocampal pyruvate kinase in ITD + RH + glucose (n = 7) and ITD + RH (n = 5‐6) groups. *P < 0.05, and ***P < 0.005

After observing significant changes in the metabolites that belong to key cellular pathways, we examined whether these changes were due to altered substrate kinetic properties of the enzymes involved in each respective pathway. Since it was practically and technically challenging to evaluate substrate kinetic properties of all enzymes of metabolic pathways affected by the exposure to RH, we evaluated substrate kinetic properties of key glycolytic enzymes (hexokinase, phosphofructokinase, and pyruvate kinase) as this pathway was significantly affected by exposure to RH. For hexokinase, we evaluated substrate kinetics for two of its substrates (ie, glucose and ATP). We did not observe any significant differences in *K*_m_ value for either substrate in both experimental groups (Figure 5B). However, the *V*_max_ for glucose was significantly lower (42%, *P* < 0.005) for ITD + RH animals (0.18 ± 0.05 mmol/L) when compared to animals belonging to ITD + RH + glucose group (0.30 ± 0.07 mmol/L) (Figure 5A). In contrast, the V_max_ for ATP was significantly higher (77%, *P* < 0.05) for ITD + RH animals (0.49 ± 0.21 mmol/L) when compared to animals belonging to the ITD + RH + glucose group (0.28 ± 0.06 mmol/L) (Figure 5B).

For phosphofructokinase, we evaluated substrate kinetics for two of its substrates (ie, fructose‐6‐phosphate and ATP). We did not observe any significant differences in K_m_ value for fructose 6‐phosphate in either experimental group (Figure 5D). However, the K_m_ value for ATP was significantly higher (292%, *P* < 0.001) for ITD + RH animals (41 ± 15 µM) when compared to animals belonging to the ITD + RH + glucose group (10 ± 3 µM) (Figure 5D). This significant increase in *K*_m_ value for ATP indicates that RH exposure decreases affinity of phosphofructokinase for ATP. The *V*_max_ for fructose 6‐phosphate was significantly lower (75%, *P* < 0.001) for ITD + RH animals (3.4 ± 2.6 mmol/L) when compared to animals belonging to the ITD + RH + glucose group (14 ± 3 mmol/L) (Figure 5C). The opposite was observed for the *V*_max_ value of ATP. The *V*_max_ for ATP was significantly higher (40%, *P* < 0.05) for ITD + RH animals (12.4 ± 1.4 mmol/L) when compared to animals belonging to the ITD + RH + glucose group (8.8 ± 2.3 mmol/L) (Figure 5C).

For pyruvate kinase, we evaluated substrate kinetics for two of its substrates (ie, phosphoenolpyruvate and ADP). The K_m_ value for phosphoenolpyruvate was significantly higher (994%, *P* < 0.005) for ITD + RH animals (580 ± 280 µM) when compared to animals belonging to ITD + RH + glucose group (53 ± 5 µM) (Figure 5F). These results indicate lower affinity of pyruvate kinase for phosphoenolpyruvate and thus lower enzyme efficiency in converting phosphoenolpyruvate into pyruvate. These results are supported by metabolomics data where we observed significantly higher levels of an upstream metabolite, 2‐phosphoglycerate (226%), in ITD + RH rats compared to ITD + RH + glucose animals. This effect may further be exacerbated during subsequent hypoglycemia exposure due to expected lower levels of substrates for glycolysis. The opposite was observed for the *K*_m_ value for ADP. The *K*_m_ value for ADP was significantly lower (84%, *P* < 0.05) for ITD + RH animals (0.39 ± 0.09 mmol/L) when compared to animals belonging to the ITD + RH + glucose group (2.4 ± 2.2 mmol/L) (Figure 5F). The *V*_max_ for phosphoenolpyruvate was significantly higher (44%, *P* < 0.005) for ITD + RH animals (17 ± 3 mmol/L) when compared to animals belonging to the ITD + RH + glucose group (12 ± 1 mmol/L) (Figure 5E). The opposite was observed for the *V*_max_ value of ADP. The *V*_max_ for ADP was lower (41%) for ITD + RH animals (14 ± 3 mmol/L) when compared to animals belonging to the ITD + RH + glucose group (23 ± 19 mmol/L) (Figure 5E). However, this difference was not statistically significant. Overall, these results demonstrate a severe impact of RH exposure on the substrate kinetic properties of glycolytic enzymes.

## 4 DISCUSSION

Both T1D and T2D patients experience frequent mild‐to‐moderate hypoglycemia throughout their life, and current research has implicated this effect on several important brain processes.^13^, ^14^, ^15^, ^16^, ^17^, ^21^ Since the brain relies mainly on glucose as a source of energy, these recurrent events of hypoglycemia are likely to affect brain metabolism leading to metabolic alterations. While earlier studies demonstrated that hypoglycemia affects several metabolomic pathways in the brain,^39^ they did not address the effect of recurrent hypoglycemia on hippocampal metabolism. Here, we employed a metabolomic approach to further elucidate the contribution of RH to hippocampal metabolism.

We first evaluated the global metabolic profile of the hippocampus in RH‐exposed ITD rats as compared to euglycemic controls (ITD + RH + Glucose). We chose to evaluate the impact of 5 episodes of hypoglycemia, per earlier studies.^15^, ^16^, ^40^ Available techniques to study metabolomics utilize analytical chemistry technologies such as nuclear magnetic resonance (NMR) and mass spectrometry (MS) to provide a comprehensive profile of the metabolites present in a biological sample.^41^ MS is more commonly used owing to its superior sensitivity, mass accuracy, and mass resolution.^42^ Metabolomics has other discernable advantages over other technologies used to study metabolism. Compared with the 100 000 transcripts and 1 000 000 proteins found in humans, there is a relatively smaller number of metabolites (~25 000) to examine.^41^ These downstream metabolites integrate genomic, transcriptomic, and proteomic variability. Precise measurement of these metabolites in disease and control conditions provides comprehensive insight into mechanistic abnormalities.^41^ Our present study and earlier studies by others reported large percentages of altered metabolites in various experimental conditions.^43^, ^44^, ^45^, ^46^, ^47^, ^48^ Based on this, it appears that metabolomic studies are more sensitive to detect the impact of various conditions on cellular functioning. We observed that prior exposure to RH leads to several differences in 65 metabolites belonging to major metabolic pathways. Our results demonstrate that RH exposure leads to metabolomic alterations in the hippocampus of ITD rats. Stress can affect glucose homeostasis and thus may also affect overall tissue metabolism.^49^ It is possible that stress produced by procedures employed in our experiments, such as insulin/glucose injections and tail puncture for blood glucose measurements, may also affect glucose homeostasis. We do not expect the impact of such stress on our conclusion, as animals belonging to both RH and RH + glucose groups were exposed to similar experimental procedures, and tissues were harvested overnight after exposure to RH or RH + glucose.

The phosphocreatine (Pcr)/creatine kinase system manages high‐energy demands of the brain. During periods of ischemia, or glutamate toxicity, Pcr converts to creatine and ATP to provide a protective effect to surrounding tissues. An association between depletion of these metabolites and severity of neurocognitive performance was shown earlier.^50^ Neuroprotective effects of creatine supplementation have been shown in rat models.^51^ We observed an increase in creatine levels in the hippocampus of RH‐exposed rats as compared to the euglycemic controls. This observed increase in creatine levels may be due to a compensatory response to RH exposure. Higher creatine levels may help maintain hippocampal energy status during the initial phase of hypoglycemia.

An earlier study evaluated the potential RH‐induced adaptation in glucose phosphorylation that may preserve glucose flux during subsequent hypoglycemia.^30^ They reported that the exposure to RH leads to increased hypothalamic glucose phosphorylation. In the present study, we did not observe increased glucose phosphorylation, indicating that prior RH exposure may not preserve glucose flux during subsequent hypoglycemia in the hippocampus. These differences may be due to a brain region‐specific effect of RH, and further studies may help identify the observed differential effects of RH on various brain regions.

Although we did not observe any statistically significant differences in levels of glucose‐6‐phosphate among both experimental groups, we did observe changes in substrate kinetic properties of hexokinase. These results indicate that although substrate kinetic properties of hexokinase were altered by the exposure to RH, these changes did not have any effect on glycolysis, at least during euglycemia. Our results also indicate that since K_m_ values for both glucose and ATP were not altered by RH exposure, changes in substrate kinetic properties of hexokinase would not have any significant impact on glycolysis during subsequent hypoglycemia.

We observed a robust decrease in the V_max_ for fructose‐6‐phosphate in ITD + RH animals (Figure 5C). This robust decrease in V_max_ indicates that phosphofructokinase may not be efficiently converting fructose 6‐phosphate into fructose 1,6‐bisphosphate. Although our metabolomics analysis did not detect levels of fructose 1,6‐bisphosphate, we did observe a nonsignificant decrease in levels of glyceraldehyde 3‐phosphate and a significant decrease in levels of dihydroxyacetone phosphate in the ITD + RH group (Figure 4A). These results indicate that the observed altered metabolome profile of glycolysis intermediates may be explained partly due to higher *K*_m_ of ATP and lower *V*_max_ of phosphofructokinase for fructose 6‐phosphate. The observation of decreased *K*_m_ for ADP and increased *V*_max_ for phosphoenolpyruvate may in fact be compensatory changes from an increased *K*_m_ for phosphoenolpyruvate and decreased *V*_max_ for ADP. It is important to note however that the data suggest that these compensatory mechanisms are not strong enough to alleviate the effects of RH on glycolysis. Considering the differential regulation of phosphofructokinase in neurons and astrocytes and the expression of a different splice variant of pyruvate kinase in neurons and astrocytes, it is possible that some of the changes we observed may be specific to astrocytes and not neurons. However, further studies dissecting the impact of hypoglycemia exposure on neuronal and astrocytic metabolism may help determine the potential differential impact of hypoglycemia on these two cell types.

We observed a significant decrease in the levels of succinate and an increase in fumarate. These results indicate that within the hippocampus of RH‐exposed rats, succinate is more efficiently converted into fumarate or it is used for other roles such as protein succinylation.^52^, ^53^ These results also indicate that mitochondrial electron transport chains may receive more electrons from FAD‐linked substrates. However, this hypothesis remains to be tested.

The levels of two of urea cycle metabolites (aspartate and citrulline) were higher in the ITD + RH group (Figure 4C). An earlier study evaluating the effect of anesthesia exposure‐induced cognitive dysfunction observed a significant increase in the levels of aspartic acid in the hippocampus of isoflurane‐treated rats, and this increase was positively correlated with the degree of cognitive dysfunction.^54^ T‐maze training of rats increases citrulline levels in the dentate gyrus and prefrontal cortex^55^ and oral administration of citrulline alleviates cerebral ischemia‐induced memory deficits.^56^ Our results indicate that the increased citrulline levels in the hippocampus of RH‐exposed rats may be a compensatory mechanism to preserve cognitive functions in the setting of the recurrent hypoglycemic insults. High levels of citrulline may be due to either increased activity of ornithine transcarbamylase (a urea cycle enzyme) or nitric oxide synthase.^57^, ^58^ From our results, it is difficult to infer a potential mechanism behind the increased citrulline levels in our experimental conditions. It is plausible that the hypoglycemia‐induced increase in cellular calcium levels may activate nitric oxide synthase, leading to increased NO and citrulline levels.^59^, ^60^ This, in turn, may also lead to increased oxidative stress. Thus, our results suggest that increased citrulline levels may indicate increased oxidative stress in hypoglycemia‐exposed animals. However, further studies are required to substantiate this hypothesis.

Prolonged glucose deprivation results in a large increase in intracellular calcium levels.^61^ Such increase in cellular calcium levels leads to the activation of calcium‐dependent phospholipase A_2_ (PLA2).^62^ Activation of PLA2 leads to the breakdown of membrane phospholipids including phosphatidylcholine resulting in increased levels of phosphatidylcholine metabolites such as phosphocholine.^63^ An earlier study demonstrated increased levels of phosphatidylcholine metabolites such as phosphocholine in cerebrospinal fluid of Alzheimer's disease patients.^63^ Increased phosphocholine levels in our experimental conditions suggest that the exposure to RH leads to PLA2 activation via increased intracellular calcium levels resulting in breakdown of membrane phospholipids leading to cellular damage in the hippocampus, and this damage may lead to RH exposure‐induced hippocampal dysfunction.

In conclusion, our results demonstrate that the exposure to RH has a profound impact on hippocampal metabolism. Our analysis also demonstrated that the changes in levels of these metabolites severely impact several metabolic pathways. These metabolic changes may be responsible for impaired hippocampal function observed following exposure to RH.

## CONFLICT OF INTEREST

The authors declare that there is no conflict of interest.
