# Supplementary material for: Exposure to recurrent hypoglycemia alters hippocampal metabolism in treated streptozotocin‐induced diabetic rats
Source: CNS Neurosci Ther. 2019 Jul 7;26(1):126–35. doi: 10.1111/cns.13186 (PMC6930817; doi:10.1111/cns.13186)
Supplement: Supplementary file 1 [file CNS-26-126-s001.docx]

**Supplementary material**

**Appendix S1. Materials and Methods**

*Induction of diabetes, insulin treatment, and induction of recurrent hypoglycemia*

All experimental procedures were carried out as per the Guide for the Care and Use of Laboratory Animals published by the National Institutes of Health and in accordance with the protocols (protocol # 14-204, 17-202) approved by the Animal Care and Use Committee of the University of Miami. Results are reported according to the ARRIVE guidelines to the best of our ability.

Male Wistar rats (3-4 months old) were made diabetic by injecting the pancreatic β cell toxin streptozotocin (58 mg / kg body weight, i.p.) (Sigma Aldrich, St. Louis, MO) (Figure 1A).^1^ To verify the induction of diabetes, blood glucose levels were measured twice a week using a portable glucose meter (Freestyle Freedom, Abbott Diabetes Care Inc., Alameda, CA, USA; detection range 20–550 mg/dl) in non-fasting rats by tail pricking. The rats having blood glucose values ≥ 250 mg/dl were included in the diabetic group. Blood glucose values greater than 550 mg/dl were assigned a value of 550 mg/dl. Insulin pellets (LinShin Canada, Inc., Ontario, Canada) were implanted subcutaneously (s.c.) after 2-3 weeks of diabetes induction.^1^ Blood glucose levels were monitored the following week and if the level was >220 mg/dl, an additional insulin pellet (or part of the pellet) was implanted to keep the glucose level in the target range (≤220 mg/dl). This group of animals was considered as insulin-treated diabetic (ITD) rats (Figure 1A).

ITD group rats were randomly divided into ITD + RH (n = 10) and ITD + RH + glucose (n = 10) groups. Rats belonging to ITD + RH or ITD + RH + glucose groups were exposed to five episodes of RH (hyperinsulinemic hypoglycemia) or RH + glucose (hyperinsulinemic euglycemia) over five consecutive days (1 episode / day), respectively. RH was induced 2-3 weeks after insulin pellet implantation. The aim of RH episodes was to maintain blood glucose levels in the moderate hypoglycemia range for three hours, by s.c. injection of Novolog (Insulin aspart, Novo Nordisk, AIS, Denmark) (Figure 1A).^1^ Glucose levels were measured at baseline and every hour during hypoglycemia. Additional insulin or 50% dextrose solution was administered by s.c. injection if the target glucose level was not achieved within the first hour of insulin injection. After three hours of hypoglycemia the rats were infused s.c. with 50% dextrose solution to raise the blood glucose to pre-hypoglycemia levels. Blood glucose levels were also measured 30 minutes post-dextrose injection (recovery) to confirm termination of the hypoglycemic episode. Animals belonging to the ITD + RH + glucose group were treated with insulin similar to the ITD + RH group. However, their glucose levels were maintained close to baseline by s.c. injection of 50% dextrose. Similar to the ITD + RH group, their blood glucose levels were measured at baseline, every hour for three hours following insulin + glucose injection, and at 30 minutes post-recovery.

One day after the last hypoglycemic exposure, the rats were euthanized by decapitation under isoflurane anesthesia (30% O_2_ / 70% N_2_O); brains were quickly excised and placed on an ice-cold dissecting tray; and, hippocampi were harvested, snap-frozen in liquid nitrogen, and stored at -80^o^ C until use for metabolomic study or enzymatic assay (Figure 1A). The total duration between decapitation and snap-freezing the hippocampus was about 2 - 3 minutes. For metabolomic analysis, the samples were shipped on dry ice to the Southeast Center for Integrated Metabolomics at the University of Florida, Gainesville, FL.

*Metabolomic studies*

Samples were analyzed in a blinded manner. The sequence of analysis was randomized using a random number generator to select the run order. The samples were extracted using pre-chilled 1:1 (v:v) methanol:water. The pre-chilled extraction mixture was added to frozen samples. After vortexing, samples were homogenized in a sonicator using 3 mm beads. Supernatant from the samples were then centrifuged and dried with liquid nitrogen. The samples were kept frozen until experimentation, then reconstituted in 0.1% formic acid in water. All samples were run in positive mode followed by negative mode. The sequence began with three blanks followed by a neat quality control (QC) and four pooled QC samples. After ten unknown samples were injected, another blank, neat QC and pooled QC samples were injected. Each pooled QC was run with data dependent/MS2 for future matching to a tandem MS library. Global metabolomics profiling was performed on a Thermo Q-Exactive Oribtrap mass spectrometer with Dionex UHPLC and autosampler. All samples were analyzed in positive and negative heated electrospray ionization with a mass resolution of 35,000 at *m/z* 200 using separate injections (2 µl for positive and 4 µl for negative). Separation was achieved on an ACE 18-pfp 100 x 2.1 mm, 2 µm column with mobile phase A as 0.1% formic acid in water and mobile phase B as acetonitrile. The flow rate was 350 µL/min with a column temperature of 25°C ^2^. Metabolite identification was performed by matching retention time (± 0.2 min) and *m/z* value (5 ppm for positive and 10 ppm for negative) to an internally curated metabolite library of 600 compounds. Samples were analyzed in both positive and negative modes. Both data sets were merged. If the same metabolite was identified in both modes, the mean value for both modes was used for further analysis.

*Metabolomic statistical, pathway, and enrichment analyses*

Metabolomic data were analyzed using MetaboAnalyst 3.0 software.^3^ Peak intensity values normalized to the lowest protein concentration in the sample set were log transformed and auto-scaled prior to statistical tests aimed at defining differential abundance of metabolites as well as multivariate analysis. One sample was ruled a significant outlier by hierarchical clustering, given that it clustered alone (with neither group), and also since Grubb’s test on several metabolites deemed that sample’s value a statistical outlier within the 95% confidence interval. A p value of <0.05 and false discovery rate of <0.1 was used as a cutoff to determine significantly altered metabolites. Principal component analysis was performed using MetaboAnalyst tool. Pathway and enrichment analyses were carried out using compound names as the input and with the following settings: pathway analysis (integrating enrichment and pathway topology) - over representation set to Fisher's Exact Test and topology to out-degree centrality; enrichment analysis 1 – pathway-associated metabolite sets based on normal metabolic pathways under basal physiological conditions. Connections between related metabolites were generated using MetaMapR^4^ in order to build a network that displays structural similarity based on a metabolite’s PubChem substructure fingerprints. The networks were then visualized using Cytoscape.^5^ All structurally annotated metabolites measured were used to generate structural connections in MetaMapR but, because not all metabolites had structural connections, not all metabolites were represented in the network.

*Tissue processing for enzyme studies*

The hippocampus was thawed on ice and, homogenized and its supernatant was collected for enzymatic assays following the manufacturer’s instructions (BioVision, Milpitas, CA, Hexokinase activity kit K789-100, Phosphofructokinase activity kit K776-100, and Pyruvate Kinase activity kit K709-100). Briefly, each hippocampus was homogenized in 500 µl hexokinase assay buffer (BioVision, Milpitas, CA, kit K789-100), kept on ice for 10 minutes with intermittent mixing, centrifuged at 10,000 x g for 5 minutes at 4^o^C. The supernatants were then collected and stored at -80^o^C until analysis. Protein concentration was measured using BioRad DC reagents (Biorad, Hercules, CA) following the manufacturer’s instructions.

*Enzyme activities and substrate kinetics studies*

Optimal protein concentration for each enzyme was first determined by plotting velocity vs protein concentration. The hexokinase enzyme assay (BioVision, Milpitas, CA, kit K789-100) was performed per manufacturer’s instructions using 1 µg protein / assay as decided based on enzyme curve results. Assay was performed over a period of 60 minutes at 25^o^C. For substrate kinetics, ATP and glucose concentrations ranged from 0.05 – 10 mM. The phosphofructokinase assay (BioVision, Milpitas, CA, kit K776-100) was performed per manufacturer’s instructions using 0.5 µg protein / assay as decided based on enzyme curve results. Assay was performed over a period of 60 minutes at 37^o^C. For substrate kinetics, ATP and fructose-6-phosphate concentrations ranged from 0.05 – 10 mM. The pyruvate kinase assay (BioVision, Milpitas, CA, kit K709-100) was also performed per manufacturer’s instructions using 0.5 µg protein / assay as decided based on enzyme curve results. Assay was performed over a period of 20 minutes at 25^o^C. For substrate kinetics, ATP and phosphoenol pyruvate concentrations ranged from 0.05 – 10 mM. Substrate kinetics data were analyzed using GraphPad Prism 5.0 software to calculate K_m_ and V_max_.

Table S1 Significantly altered, structurally annotated metabolites in ITD + RH group.

| **Metabolite name** | **Fold change** | ***p*-value** | **FDR** |
| --- | --- | --- | --- |
| N-Acetyl-Hexosamine | 1.4262 | 0.0002 | 0.0190 |
| Glyceric acid | 2.1723 | 0.0007 | 0.0283 |
| ADP | 0.2782 | 0.0007 | 0.0283 |
| Sarcosine/Beta-Alanine | 1.1955 | 0.0034 | 0.0409 |
| N-Methyl-D-Aspartic Acid | 1.2696 | 0.0038 | 0.0409 |
| D-Glucosamine 6-Phosphate | 1.4606 | 0.0039 | 0.0409 |
| Aldopentose | 1.3473 | 0.0040 | 0.0409 |
| 3-Hydroxy-3-Methylglutarate | 1.5162 | 0.0041 | 0.0409 |
| Betaine | 18.0020 | 0.0044 | 0.0409 |
| Malate | 1.4854 | 0.0045 | 0.0409 |
| Dihydroxyacetone Phosphate | 0.5310 | 0.0046 | 0.0409 |
| 2-Amino-2-Methylpropanoate | 1.3129 | 0.0050 | 0.0409 |
| 3-Ureidopropionate | 1.5936 | 0.0051 | 0.0409 |
| Guanosine 5'-Monophosphate | 0.5833 | 0.0055 | 0.0409 |
| Fumaric Acid | 1.6493 | 0.0056 | 0.0409 |
| Phosphocholine | 1.3317 | 0.0061 | 0.0409 |
| R-Malate | 1.3548 | 0.0063 | 0.0409 |
| Threonine/Homoserine | 1.3482 | 0.0067 | 0.0409 |
| L-2-Phosphoglyceric acid | 3.2649 | 0.0068 | 0.0409 |
| Xanthine | 1.3503 | 0.0075 | 0.0429 |
| Creatinine | 1.4743 | 0.0081 | 0.0445 |
| 1-Aminocyclopropane-1-Carboxylate | 1.2109 | 0.0094 | 0.0487 |
| Aspartate | 1.3958 | 0.0097 | 0.0487 |
| Thymine | 1.7443 | 0.0104 | 0.0498 |
| L-Methionine | 1.5582 | 0.0115 | 0.0509 |
| L-Cysteine | 1.6964 | 0.0122 | 0.0509 |
| L-Valine | 1.6011 | 0.0123 | 0.0509 |
| Hypoxanthine | 1.4090 | 0.0125 | 0.0509 |
| Alanine/Sarcosine | 1.3942 | 0.0130 | 0.0509 |
| N-Acetyl-L-Aspartic acid | 1.2379 | 0.0135 | 0.0509 |
| Cys-Gly | 1.8610 | 0.0137 | 0.0509 |
| Gluconic acid/D-Gulonic acid Gama-Lactone | 1.6586 | 0.0142 | 0.0509 |
| Adenosine | 0.3173 | 0.0148 | 0.0515 |
| Urate | 1.4615 | 0.0165 | 0.0527 |
| L-Tyrosine | 1.4961 | 0.0171 | 0.0527 |
| Nicotinamide | 1.3703 | 0.0177 | 0.0527 |
| Phenylalanine-HCOOH | 1.5150 | 0.0179 | 0.0527 |
| L-Leucine-D10 | 0.9002 | 0.0180 | 0.0527 |
| Phenylalanine | 1.5047 | 0.0185 | 0.0527 |
| L-Isoleucine | 1.4278 | 0.0186 | 0.0527 |
| L-Proline | 1.3415 | 0.0189 | 0.0527 |
| S-5'-Adenosyl-L-Homocysteine | 1.4401 | 0.0194 | 0.0527 |
| 5-Oxo-L-Proline | 1.4233 | 0.0197 | 0.0527 |
| 3-Sulfino-L-Alanine | 1.6272 | 0.0202 | 0.0527 |
| 4-Guanidinobutanoate | 1.3432 | 0.0223 | 0.0559 |
| Tryptophan | 1.4809 | 0.0229 | 0.0559 |
| Leucine | 1.4001 | 0.0235 | 0.0559 |
| Tryptophan-NH3 | 1.4771 | 0.0236 | 0.0559 |
| Adenosine 5'-Monophosphate | 0.5206 | 0.0238 | 0.0559 |
| LL-2,6-Diaminoheptanedioate | 1.5266 | 0.0243 | 0.0559 |
| Arachidonic Acid 204 | 4.3811 | 0.0253 | 0.0570 |
| L-Valine/5-Aminopentanoate/L-Norvaline | 1.3356 | 0.0261 | 0.0571 |
| Caffeine-D3 | 0.8936 | 0.0263 | 0.0571 |
| Glyceraldehyde/Lactate | 1.3781 | 0.0275 | 0.0582 |
| Creatine | 1.2396 | 0.0278 | 0.0582 |
| N-Acetyl-DL-Glutamic Acid | 1.4095 | 0.0322 | 0.0660 |
| Succinic Acid-13C6 | 0.7896 | 0.0383 | 0.0772 |
| Pyridoxal | 1.2964 | 0.0401 | 0.0795 |
| Docosahexaenoic Acid 226 | 3.5591 | 0.0409 | 0.0795 |
| 5,6-Dihydrouracil | 1.3238 | 0.0415 | 0.0795 |
| Uracil | 1.4392 | 0.0431 | 0.0812 |
| Methyl Beta-D-Galactoside | 0.7661 | 0.0442 | 0.0820 |
| Citrulline | 1.3448 | 0.0468 | 0.0854 |
| Inosine 5'-Monophosphate | 0.5283 | 0.0485 | 0.0861 |
| Citrate | 1.3848 | 0.0487 | 0.0861 |

*Data from non-targeted primary metabolomics analysis of ITD + RH + Glucose and ITD + RH hippocampus. Only structurally annotated metabolites are presented. Fold change is with respect to ITD + RH + Glucose group.

**References**

1. Dave KR, Tamariz J, Desai KM, et al. Recurrent hypoglycemia exacerbates cerebral ischemic damage in streptozotocin-induced diabetic rats. *Stroke.* 2011;42(5):1404-1411.

2. Ulmer CZ, Yost RA, Chen J, Mathews CE, Garrett TJ. Liquid Chromatography-Mass Spectrometry Metabolic and Lipidomic Sample Preparation Workflow for Suspension-Cultured Mammalian Cells using Jurkat T lymphocyte Cells. *J Proteomics Bioinform.* 2015;8(6):126-132.

3. Xia J, Wishart DS. Using MetaboAnalyst 3.0 for Comprehensive Metabolomics Data Analysis. *Curr Protoc Bioinformatics.* 2016;55:14 10 11-14 10 91.

4. Grapov D, Wanichthanarak K, Fiehn O. MetaMapR: pathway independent metabolomic network analysis incorporating unknowns. *Bioinformatics.* 2015;31(16):2757-2760.

5. Kohl M, Wiese S, Warscheid B. Cytoscape: software for visualization and analysis of biological networks. *Methods Mol Biol.* 2011;696:291-303.
